# Structure of the unique SEFIR domain from human interleukin 17 receptor A reveals a composite ligand-binding site containing a conserved α-helix for Act1 binding and IL-17 signaling

**DOI:** 10.1107/S1399004714005227

**Published:** 2014-04-30

**Authors:** Bing Zhang, Caini Liu, Wen Qian, Yue Han, Xiaoxia Li, Junpeng Deng

**Affiliations:** a Department of Biochemistry and Molecular Biology, Oklahoma State University, Stillwater, OK 74078, USA; b Department of Immunology, Lerner Research Institute, Cleveland Clinic, 9500 Euclid Avenue, Cleveland, OH 44195, USA

**Keywords:** SEFIR domain, interleukin 17 receptor A, Act1 binding, IL-17 signaling

## Abstract

Interleukin 17 (IL-17) cytokines play a crucial role in mediating inflammatory and autoimmune diseases. A unique intracellular signaling domain termed SEFIR is found within all IL-17 receptors (IL-17Rs) as well as the key adaptor protein Act1. SEFIR-mediated protein–protein interaction is a crucial step in IL-17 cytokine signaling. Here, the 2.3 Å resolution crystal structure of the SEFIR domain of IL-17RA, the most commonly shared receptor for IL-17 cytokine signaling, is reported. The structure includes the complete SEFIR domain and an additional α-helical C-terminal extension, which pack tightly together to form a compact unit. Structural comparison between the SEFIR domains of IL-17RA and IL-17RB reveals substantial differences in protein topology and folding. The uniquely long insertion between strand βC and helix αC in IL-17RA SEFIR is mostly well ordered, displaying a helix (αCC′_ins_) and a flexible loop (CC′). The DD′ loop in the IL-17RA SEFIR structure is much shorter; it rotates nearly 90° with respect to the counterpart in the IL-17RB SEFIR structure and shifts about 12 Å to accommodate the αCC′_ins_ helix without forming any knots. Helix αC was identified as critical for its interaction with Act1 and IL-17-stimulated gene expression. The data suggest that the heterotypic SEFIR–SEFIR association *via* helix αC is a conserved and signature mechanism specific for IL-17 signaling. The structure also suggests that the downstream motif of IL-17RA SEFIR together with helix αC could provide a composite ligand-binding surface for recruiting Act1 during IL-17 signaling.

## Abstract 2

Crystal structure of the SEFIR domain from human IL-17 receptor A provides new insights into IL-17 signaling.

## 1. Introduction   

Members of the IL-17 cytokine family (IL-17A to IL-17F) are important regulators of both innate and adaptive immune responses. However, the strong inflammatory response overstimulated by IL-17 contributes to the pathogenesis of various human and animal diseases, such as rheumatoid arthritis, multiple sclerosis (MS), experimental autoimmune encephalomyelitis (EAE; a mouse model of MS) and allergen-induced pulmonary inflammation (Ye *et al.*, 2001 ▶; Nakae *et al.*, 2002 ▶; Iwakura & Ishigame, 2006 ▶; Schnyder-Candrian *et al.*, 2006 ▶; Conti *et al.*, 2009 ▶; Gonzalez-García *et al.*, 2009 ▶; Hu *et al.*, 2011 ▶). The IL-17 receptor (IL-17R) family comprises five members (IL-17RA to IL-17RE) with considerable sequence divergence, which are all membrane-bound proteins containing single transmembrane domains. IL-17Rs contain fibronectin type III (FnIII) domains within the extracellular portion and a unique structural motif within the cytoplasmic tails termed the SEFIR [SEF (similar expression to fibroblast growth factor genes) and IL-17R] domain (Novatchkova *et al.*, 2003 ▶). SEFIR domains share limited homology with the Toll/IL-1 receptor (TIR) domains of Toll-like receptors (TLRs) and IL-1R (Novatchkova *et al.*, 2003 ▶). Proteins containing SEFIR and TIR domains together constitute the STIR (SEFIR/TIR) domain superfamily. NF-κB activator 1 [Act1; also known as connection to I-κB kinase and stress-activated protein kinase (CIKS); Leonardi *et al.*, 2000 ▶] is also a member of the SEFIR protein family and is an essential component in the signaling of several IL-17 cytokines (Chang *et al.*, 2006 ▶; Qian *et al.*, 2007 ▶; Claudio *et al.*, 2009 ▶; Swaidani *et al.*, 2009 ▶). Although the exact stoichiometry of the complex of IL-17R and Act1 in IL-17 signaling is not known, SEFIR-mediated homotypic and heterotypic interactions among IL-17Rs and Act1 play a crucial role in IL-17R signaling. The crystal structure of the IL-17RB SEFIR domain has recently been determined, which revealed a unique knot in protein folding (Zhang *et al.*, 2013 ▶). Structural elements unique to SEFIR and distinct from TIR domains have been identified, including a long CC′ insertion (between strand βC and helix αC) and a rigid short helix αB′. It has been shown that the αC helices of the SEFIR domains from IL-17RB SEFIR and Act1 are critical for their heterotypic interactions, while helix αB′ of Act1 is responsible for its self-association. The study provided the first structural view of the IL-17 receptor intracellular signaling, unraveling the mechanism for the specificity of the SEFIR *versus* the TIR domain in their respective signaling pathways. However, questions still remain for the SEFIR domains from other IL-17 receptors owing to low pairwise sequence identity (less than 20%). In particular, IL-17RA appears to be unique among all IL-17Rs and contains the longest intracellular domain, which extends well beyond its SEFIR domain. In addition, it has been shown that IL-17RA signaling requires not only its SEFIR domain but also a short C-terminal extension of about 100 amino acids (Onishi *et al.*, 2010 ▶). This tandem structural motif was termed the SEFEX domain and was found to be important for both binding Act1 and IL-17 cytokine signaling (Onishi *et al.*, 2010 ▶). The fact that IL-17RA SEFIR alone is not sufficient for binding Act1 and IL-17 signaling suggests that it could be structurally unique when compared with the SEFIR domains of other IL-17Rs and Act1.

Since IL-17 is an important regulator for inflammation, blocking IL-17 signaling is a promising approach for the treatment of autoimmune diseases. For example, brodalumab, an antibody against IL-17RA for the treatment of psoriasis, is currently in phase 2 clinic trials (Papp *et al.*, 2012 ▶). Given the importance of SEFIR–SEFIR interactions in IL-17 cytokine signaling, a better mechanistic understanding of these interactions is crucial for the development of new and improved therapeutics for the treatment of IL-17 cytokine-mediated inflammatory diseases. Here, we report the crystal structure of a fragment from the IL-17RA intracellular signaling domain including its SEFIR domain and a short C-extension at 2.3 Å resolution. The structure displays large differences in protein topology and conformation with respect to the structure of IL-17RB SEFIR. Our structural and functional analyses again reveal that helix αC of IL-17RA is important for its binding to Act1 and IL-17 cytokine signaling. This suggests that the heterotypic SEFIR–SEFIR association *via* helix αC is a conserved and signature mechanism specific for IL-17 cytokine signaling. The structure of IL-17RA SEFIR also suggests that the downstream motif of IL-17RA SEFIR could fold back close to helix αC of SEFIR, together providing a composite ligand-binding surface that is necessary for recruiting Act1 during IL-17 cytokine signaling.

## 2. Materials and methods   

### 2.1. Protein expression and purification   

A fragment of human IL-17RA (amino-acid residues 351–597; termed IL-17RA SEFIR) containing the entire SEFIR domain was cloned into a modified pET-28 vector with an N-terminal 6×His tag. The IL-17RA SEFIR domain was expressed and purified using a similar protocol as described previously (Krumm *et al.*, 2008 ▶). The N-terminal 6×His tag was removed using *Tobacco etch virus* (TEV) protease. The purified protein was concentrated to 20 mg ml^−1^ and crystallized in a condition consisting of 0.1 *M* HEPES pH 7.5, 3.5 *M* sodium nitrate after about a week at room temperature. However, the crystals did not diffract at the synchrotron. The purified protein was then subjected to limited proteolysis by treatment with chymotrypsin, and was subsequently further purified by size-exclusion chromatography on a Superdex S200 column (GE Healthcare). The purified chymotrypsin-processed protein sample displayed a single peak on the S200 sizing profile with an estimated molecular mass of 28 kDa, which is nearly the same as the intact protein sample. Selenomethionine (SeMet)-substituted IL-17RA SEFIR domain was expressed in M9 minimal medium supplemented with amino acids as described by Van Duyne *et al.* (1993 ▶) and purified similarly to the native protein using the procedures described above. For optimal reproducibility of crystallization, all purified proteins were flash-frozen and stored at −80°C until usage (Deng *et al.*, 2004 ▶).

### 2.2. Crystallization, data collection and structure determination   

The chymotrypsin-treated IL-17RA SEFIR crystallized in a condition consisting of 0.1 *M* MES pH 6.0, 5–10% PEG 10 000. 20% glycerol was added to the mother liquor as a cryoprotectant. A set of data was collected from an SeMet-substituted protein crystal on beamline 19-ID at the Advanced Photon Source, Argonne National Laboratory, USA. The structure was solved by the single-wavelength anomalous dispersion method using *HKL*-3000 (Minor *et al.*, 2006 ▶). A nearly complete model was constructed from the experimental phases obtained from the SeMet crystal data. This model was used to solve the native structure by the molecular-replacement method using *Phaser* (McCoy *et al.*, 2007 ▶). *PHENIX* (Adams *et al.*, 2010 ▶) was used for refinement and *Coot* (Emsley *et al.*, 2010 ▶) was used for iterative manual model building. Translation, libration and screw-rotation displacement (TLS) groups used in the refinement were defined by the *TLMSD* server (Painter & Merritt, 2006 ▶). The current model has good geometry and refinement statistics (Table 1 ▶). The final structure was refined to 2.3 Å resolution and the crystallographic *R*_work_ and *R*_free_ values are 18.9 and 24.1%, respectively. The final protein model has 96.3% of all residues residing in the most favored region of the Ramachandran plot and 3.7% in additionally allowed regions, as calculated by the *MolProbity* server (Chen *et al.*, 2010 ▶). All molecular-graphics figures were generated with *PyMOL* (DeLano, 2002 ▶). Atomic coordinates and structure factors have been deposited in the Protein Data Bank (http://www.rcsb.org; PDB code 4nux).

### 2.3. Cell culture, plasmids and antibodies   

Mouse embryonic fibroblasts (MEFs) were maintained in Dulbecco’s modified Eagle’s medium (DMEM) supplemented with 10% fetal bovine serum (FBS), penicillin G (100 µg ml^−1^), and streptomycin (100 µg ml^−1^). Complementary DNA (cDNA) encoding Myc-tagged mouse IL-17RA and its point mutants were subcloned into the plasmid pMSCV-IRES-GFP. Mouse Act1 antibody was generated by Invitrogen custom antibody services. Mouse Myc antibody was purchased from Cell Signaling.

### 2.4. Retroviral infection and co-immunoprecipitation   

For reconstitution assays in MEFs, cells were infected by retroviral supernatant as described previously (Qian *et al.*, 2007 ▶). For co-immunoprecipitations, cell extracts were incubated with antibody (1 µg) and protein A beads (20 µl). After overnight incubation, beads were washed four times with lysis buffer, resolved by SDS–PAGE, and analyzed by Western blotting according to standard procedures.

### 2.5. Real-time polymerase chain reaction assays   

Total RNA was extracted with TRIzol reagent according to the manufacturer’s instructions. The cDNAs were synthesized and real-time reverse transcription polymerase chain reaction (RT-PCR) assays were performed as described previously (Qian *et al.*, 2007 ▶).

## 3. Results   

### 3.1. The structure of IL-17RA SEFIR   

**Figure 1:**
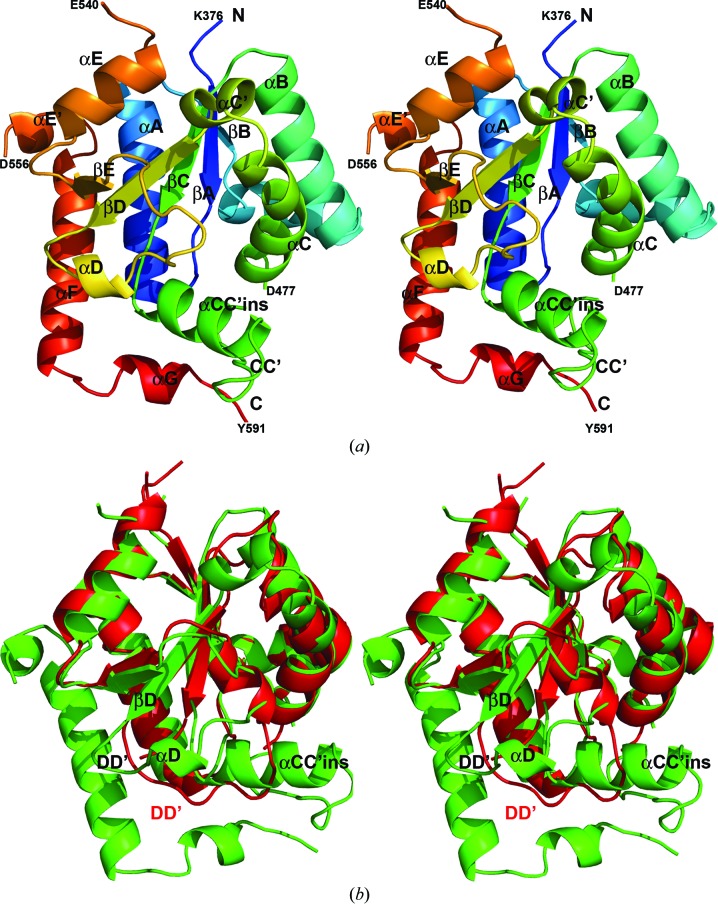
The unique structure of IL-17RA SEFIR. (*a*) Stereoview of the IL-17RA SEFIR domain. The secondary structures are shown as rainbow-colored ribbons and are labeled. (*b*) Pairwise comparison with IL-17RB SEFIR. Depicted is a stereoview of the superimposed structures of the SEFIR domains from IL-17RA (green) and IL-17RB (red). The secondary structures with the greatest topological and conformational differences are labeled.

The structure of IL-17RA SEFIR displays a compact globular architecture comprised of a five-stranded (βA–βE) parallel β-sheet that is wrapped by 11 helices (αA, αB′, αB, αCC′_ins_, αC, αC′, αD, αE, αE′, αF and αG; Fig. 1 ▶ *a*) with loops of various lengths connecting the secondary structures (the individual loop that connects the β-strand and its next neighboring helix is named after the names of the secondary structures, *i.e.* the DD′ loop connects strand βD and helix αD). The structure is well defined except for two flexible loops, the electron densities of which are not observable. One is a six-amino-acid short linker (residues 471–476) within the CC′ loop. The other is a 14-amino-acid linker (residues 541–555) connecting helices αE and αE′. Besides the complete SEFIR domain (residues 376–540) of IL-17RA, the structure reported here additionally includes a short C-terminal extension (residues 556–591) of about 35 amino acids. Extending from the C-terminus of the SEFIR domain, the C-terminal fragment (residues 556–591) forms additional α-helices (αE′, αF and αG; Fig. 1 ▶ *a*). The longer helix αF is sandwiched by two short helices, αE′ and αG, in a Z-shaped crank shape. This predominantly helical C-extension of SEFIR packs tightly against helices αA and CC′_ins_ and loops CC′, DD′ and EE′ through extensive van der Waals interactions, stabilizing the overall architecture.

### 3.2. IL-17RA SEFIR *versus* IL-17RB SEFIR   

**Figure 2:**
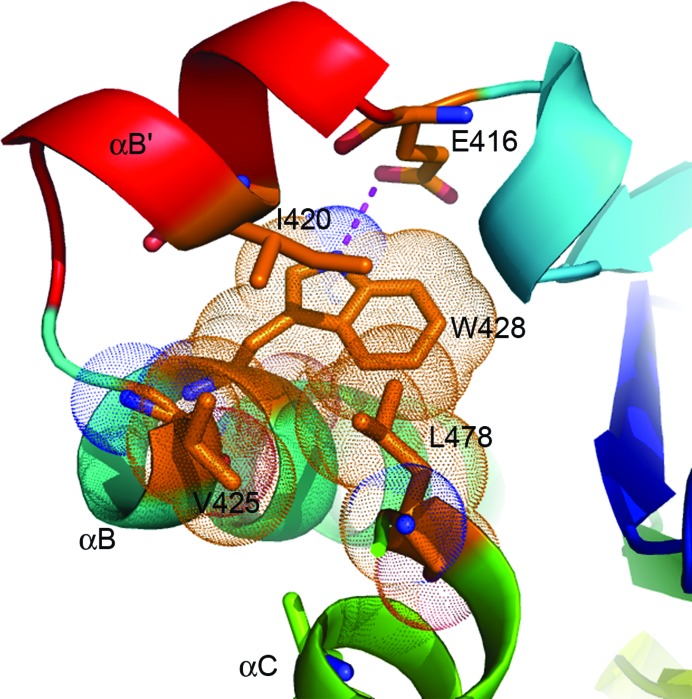
Helix αB′ is tethered to helices αB and αC *via* hydrophobic and hydrogen-bonding interactions. The side chains of the hydrophobic residues are shown as sticks with dotted spheres showing the van der Waals radius for each atom. The hydrogen bond between N^∊1^ of Trp428 and O^∊1^ of Glu416 is shown as a purple dashed line.

**Figure 3:**
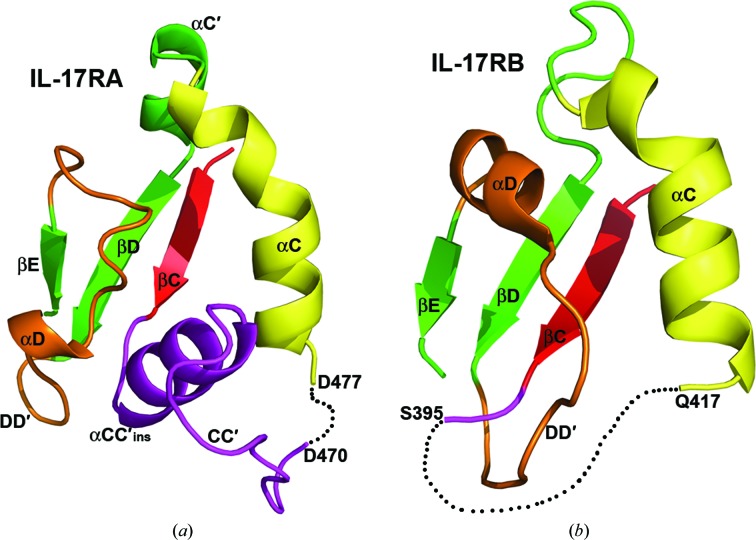
IL-17RA and IL-17RB SEFIR domains adopt different topologies. Depicted are partial structures of the IL-17RA (left) and IL-17RB (right) SEFIR domains. For clarity, parts of the SEFIR structures are omitted. The black dashed lines represent disordered loops that are not observed in the structures. Notice that strand βC (red) and helix αC (yellow) in IL-17RA SEFIR are connected by a long insertion containing αCC′_ins_ and the CC′ loop (purple) without forming a knot. In contrast, the corresponding fragment in IL-17RB SEFIR is largely disordered and connects strand βC (red) and helix αC (yellow) in a knot topology.

The structure of IL-17RA SEFIR partially resembles that of IL-17RB SEFIR (Zhang *et al.*, 2013 ▶), with an r.m.s.d. of 1.82 Å over 121 aligned C^α^ atoms (Fig. 1 ▶ *b*). The central five-stranded parallel β-sheets of the two structures superimpose very well with little conformational change. Helices αA, αB′, αB, αC and αE also align very well between the two structures. Similar to the observation in the structure of IL-17RB SEFIR, the structure of IL-17RA SEFIR also contains a short helix αB′ (residue 413–422) that links helix αB and strand βB and is nearly perpendicular to helix αB (Fig. 1 ▶). Helix αB′ is also tethered to helices αB and αC through hydrophobic inter­actions in a similar way as observed in the IL-17RB SEFIR structure. Specifically, residue Ile420 on helix αB′ is associated with a hydrophobic platform comprised of residues Val425 and Trp428 on helix αB and Leu478 on helix αC (Fig. 2 ▶). In addition, residue Trp428 on helix αB is hydrogen bonded to Glu416 on αB′, which further stabilizes and locks the αB′ and αB helices into a rigid conformation. Despite these structural similarities, the two SEFIR structures also display significant differences in protein folding and conformation. In the IL-17RA SEFIR structure there is a short helix (αC′; residues 493–498) between the kinked helix αC and strand βD (Figs. 1 ▶ *a* and 3 ▶). The equivalent part in the IL-17RB SEFIR structure is a loop. In contrast to the complete disorder in the structure of IL-17RB SEFIR, the unique and long insertion between strand βC and helix αC in the IL-17RA SEFIR structure is mostly well ordered, displaying a helix (αCC′_ins_) and a flexible loop (CC′) (Figs. 1 ▶ and 3 ▶). This observation is consistent with our secondary-structure prediction (Zhang *et al.*, 2013 ▶). Helix αCC′_ins_ is nearly perpendicular to both strand βC and helix αC in the IL-17RA SEFIR structure. The most striking observation when comparing the two SEFIR structures is the absence of the knot feature in IL-17RA SEFIR (Fig. 3 ▶). There is a large conformational difference in the secondary structures connecting strands βD and βE between the two crystal structures. In the IL-17RA SEFIR structure the much shorter DD′ loop together with the short helix αD rotate up almost 90° with respect to the much longer DD′ loop in IL-17RB SEFIR structure and shift about 12 Å to accommodate the αCC′_ins_ helix (Figs. 1 ▶ *b* and 3 ▶). As a consequence, the topology of IL-17RA SEFIR is completely different from that of IL-17RB SEFIR and lacks a knot. In addition, the structure of IL-17RB SEFIR does not contain any C-terminal extensions beyond helix αE as observed in the IL-17RA SEFIR structure (Fig. 1 ▶).

### 3.3. Helix αC is critical for IL-17RA signaling   

**Figure 4:**
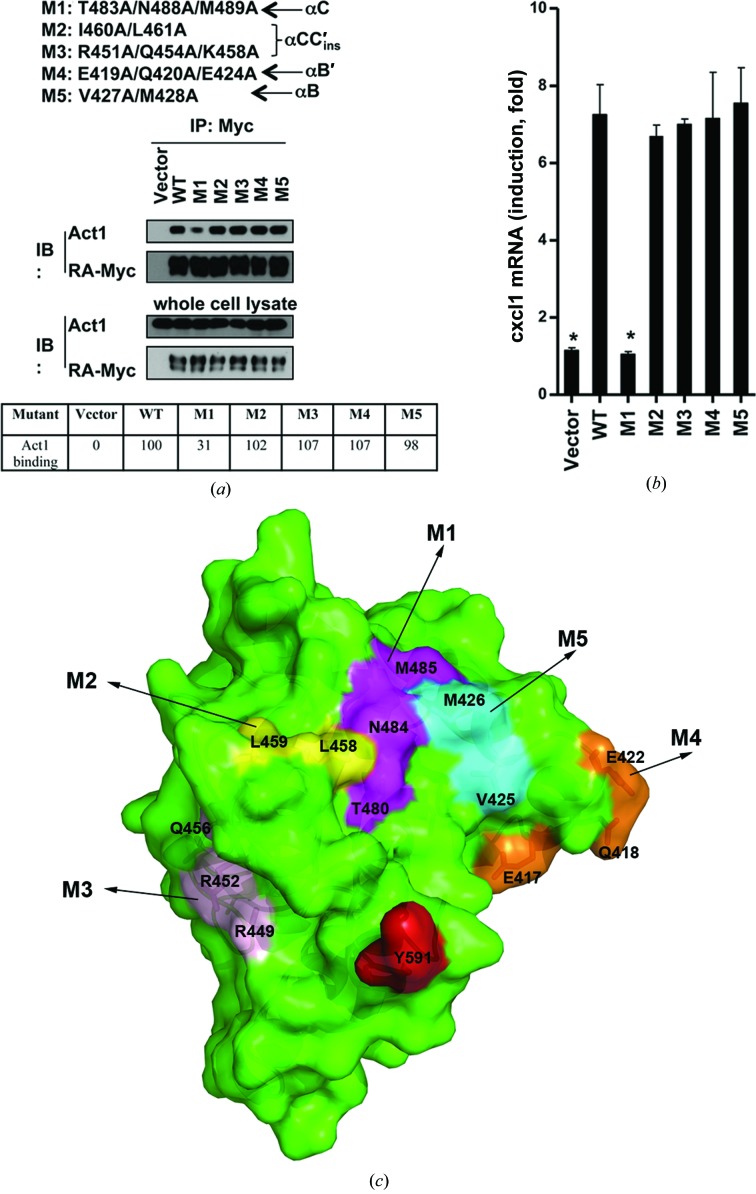
Helix αC of IL-17RA SEFIR is key to function. (*a*) IL-17RA-deficient MEFs were reconstituted with empty vector and Myc-tagged mouse RA mutants by retroviral infection, after which the cells were treated with IL-17 for 10 min followed by immunoprecipitation with anti-Myc antibody and Western blot analysis with the indicated antibodies. The densities of Act1 bands from the immunocomplexes and whole cell lysate were quantified using *ImageJ* and their ratios were normalized with respect to the wild-type sample and shown as Act1 binding. (*b*) The same cells as in (*a*) were treated with IL-17 for 3 h. The abundances of *cxcl1* mRNAs were measured by real-time RT-PCR and the induction (fold) of mRNA was calculated as the ratio of the amount of mRNA in the treated sample compared with that in the untreated sample. Data are means ± SEMs from three experiments. An asterisk indicates *p* < 0.05 (a difference from the wild-type sample). (*c*) The functionally defective mutations are located on the surface of helix αC. The IL-17RA SEFIR structure is shown in green as a surface representation. The surfaces of the human IL-­17RA SEFIR structure corresponding to the five mouse mutation sites are colored magenta (M1; T480/N484/M485), yellow (M2; L458/L459), light pink (M3; R449/R452/Q456), orange (M4; E417/Q418/E422) and cyan (M5; V425/M426). The C-terminal end of helix αG (residue Tyr591) is shown in red. Note that Tyr591 is oriented in the same direction as the surface of helix αC.

The adaptor protein Act1 is a key component in IL-17 cytokine signaling (Chang *et al.*, 2006 ▶, 2011 ▶; Qian *et al.*, 2007 ▶; Claudio *et al.*, 2009 ▶; Swaidani *et al.*, 2009 ▶; Ramirez-Carrozzi *et al.*, 2011 ▶; Song *et al.*, 2011 ▶). It has been implied that IL-17RA may be a common receptor unit for IL-17 cytokines. Act1 mediates IL-17RA signaling in a SEFIR-dependent manner (Claudio *et al.*, 2009 ▶; Swaidani *et al.*, 2009 ▶) *via* the heterodimerization of Act1 and IL-17RA. Deletion of the SEFIR domain of either protein abolished their interaction. To identify residues in IL-17RA SEFIR that are key to its function, based on the structure of IL-17RA SEFIR we generated five mutants of mouse IL-17RA SEFIR and performed reconstitution and co-immunoprecipitation experiments in IL-17RA^−/−^ MEFs. Since we have previously shown that helix αC of the SEFIR domains of IL-17RB and Act1 is important for SEFIR-mediated protein–protein interactions and IL-17 signaling (Liu *et al.*, 2011 ▶; Zhang *et al.*, 2013 ▶), we decided to test surface residues of IL-17RA SEFIR located on the secondary structures that are in the vicinity of helix αC. The mutations are grouped into the following four regions on the surface of IL-17RA SEFIR (Fig. 4 ▶): αC, M1 (T483A/N488A/M489A); αCC′_ins_, M2 (I460A/L461A) and M3 (R451A/Q454A/K458A); αB′, M4 (E419A/Q420A/E424A); αB, M5 (V427A/M428A). We found that only mutations in helix αC (M1; T483A/N488A/M489A) substantially decreased the inter­action of IL-17RA with Act1 and abolished IL-17A-induced gene expression (Figs. 4 ▶ *a* and 4 ▶ *b*). In contrast, all other mutations had little impact on the interaction of IL-17RA with Act1 and IL-17A signaling. The results here implicate that the αC helix of IL-17RA is the most critical region within the SEFIR domain for its heterotypic interaction with Act1 and is essential for IL-17A signaling.

## 4. Discussion   

We have determined the 2.3 Å resolution crystal structure of the SEFIR domain of IL-17RA, the most commonly shared receptor subunit for IL-17 cytokine-mediated immune responses. The current study provides key information on the unique structural module that is the signature for IL-17 intracellular signaling. The structure of IL-17RA SEFIR revealed substantial differences in protein folding and conformation when compared with the recently reported structure of IL-17RB SEFIR (Zhang *et al.*, 2013 ▶). Our structural and functional analysis of IL-17RA SEFIR identified helix αC as a critical structural motif for heterotypic SEFIR–SEFIR interactions between Act1 and IL-17RA. Specifically, we show that alanine substitutions of residues Thr483, Asn488 and Met489 that are located on the surface of helix αC of mouse IL-17RA SEFIR impaired its interaction with Act1 as well as IL-17A-induced gene expression. Our data suggest that helix αC is a conserved hot spot for heterotypic SEFIR-mediated protein interactions. The structure of IL-17RA SEFIR reveals that its structural stability is dependent on the helical C-extension, which is a distinct feature specific to IL-17RA. The structure also suggests that a structural motif further downstream of SEFIR could cluster spatially with helix αC and provide a composite surface for recruiting Act1 during IL-17 signaling.

The crystal structure of IL-17RB SEFIR has recently been reported, which provided a fresh look at the unique signaling module responsible for IL-17 signaling and provided important structural insights (Zhang *et al.*, 2013 ▶). However, the low pairwise sequence identity (less than 20%) among IL-17R SEFIR domains warrants further structural and functional studies on other SEFIR domains. This is particularly important for IL-17RA SEFIR because it is shared among essentially all IL-17 receptor complexes for signaling (Gaffen, 2009 ▶; Chang & Dong, 2011 ▶). Comparison of the structures of the IL-17RA and IL-17RB SEFIR domains revealed that both SEFIR domains share a conserved set of secondary structures, especially the central five-stranded parallel β-sheet and helices αA, αB′, αB, αC and αE, forming a compact structural core (Fig. 1 ▶ *b*). Similar to our observations for the structure of IL-17RB SEFIR, the short helix αB′ between βB and αB is also tethered to helices αB and αC through hydrophobic interactions and a conserved hydrogen bond between Trp428 and Glu416 in the IL-17RA SEFIR structure. These observations suggest that helix αB′ adopts a stable and relatively rigid conformation that distinguishes the SEFIR domain from a TIR domain (Zhang *et al.*, 2013 ▶). We previously showed that helix αB′ of Act1 was not essential for its heterotypic SEFIR–SEFIR interactions, since deletion of this segment in Act1 did not have a significant impact on its interaction with IL-17RA (Liu *et al.*, 2011 ▶). However, we showed that helix αB′ in Act1 SEFIR is instead essential for its homotypic SEFIR–SEFIR interactions, since deletion of helix αB′ in the Act1 SEFIR domain greatly reduced its self-association and IL-17 cytokine-mediated gene expression (Zhang *et al.*, 2013 ▶). The functional role of helix αB′ in IL-17RA SEFIR is not clear. However, alanine substitutions of charged and polar residues on the surface of helix αB′ did not show any significant impact on its binding to Act1 and IL-17A signaling (Figs. 4 ▶ *a* and 4 ▶ *b*). Therefore, helix αB′ seems to represent a conserved structural feature among all SEFIR-containing proteins, but may possess varying functional roles. In addition, we have previously shown helix αC of both Act1 and IL-17RB SEFIR domains to be a key structural element and a ‘hot spot’ for heterotypic SEFIR-mediated protein interactions. Surface mutations on helix αC in both the Act1 and IL-17RB SEFIR domains disrupted their respective interactions with IL-17RA and Act1, and abolished IL-17 cytokine-stimulated gene expression (Liu *et al.*, 2011 ▶; Zhang *et al.*, 2013 ▶). This led to our hypothesis that heterotypic SEFIR–SEFIR association *via* helix αC is a conserved and signature mechanism specific for IL-17R signaling. The structural and functional data presented here on IL-17RA SEFIR support this hypothesis.

Despite sharing many structural features with IL-17RB SEFIR, the IL-17RA SEFIR structure displays substantial differences in protein folding and conformation. First of all, IL-17RA SEFIR is topologically different from IL-17RB SEFIR and does not contain any knot features. Secondly, the unique signature insertion between βC and αC is well ordered in the IL-17RA SEFIR structure, in comparison to the complete disorder in the IL-17RB SEFIR structure that is presumably owing to significant flexibility. Although our mutagenesis studies on the surface of helix αCC′_ins_ showed negligible effects on the binding to Act1 and IL-17-stimulated gene expression, αCC′_ins_ is tightly sandwiched between helices αC and αD and contributes to the overall structural integrity of IL-17RA SEFIR (Figs. 1 ▶ *a* and 3 ▶). Besides the drastic difference in topology, the greatest conformational changes observed between the two SEFIR structures lie in the DD′ loop and helix αD. The DD′ loop in IL-17RA SEFIR is much shorter compared with that in IL-17RB SEFIR. Together with the short helix αD, it rotates almost 90° with respect to the IL-17RB SEFIR structure and shifts by more than 12 Å, making sufficient space to accommodate the αCC′_ins_ helix.

IL-17RA is unique among all IL-17Rs in containing the longest intracellular domain, which extends well beyond the SEFIR domain. Although the SEFIR domain of IL-17RA is critical for function, it has also been shown that its C-terminal extension of about 100 amino acids is additionally required for complete Act1 binding and IL-17 signaling (Onishi *et al.*, 2010 ▶). This tandem module was termed the SEFEX domain and is uniquely present in the IL-17RA intracellular domain. The fact that IL-17RA SEFIR alone is not sufficient for binding Act1 and for IL-17 signaling suggests that it could be structurally unique when compared with the SEFIRs from other IL17Rs and Act1. Indeed, the IL-17RA SEFIR structure contains not only the complete SEFIR domain but also a short helical C-terminal extension. The additional helices from the C-terminal ­extension of the SEFIR are tethered to the protein core and provide important stabilization for the structural folding. Therefore, the SEFIR domain of IL-17RA itself is structurally and functionally distinct compared with other SEFIRs lacking C-terminal extensions. Onishi and coworkers showed that a downstream region between residues 625 and 645 is also critical for IL-17 signaling, and named the tandem of SEFIR and its C-terminal extension the SEFEX domain. Although the structure of IL-17RA SEFIR does not extend beyond residue 591, it provides insights into a possible role of the SEFEX domain. Specifically, the C-terminal helix αG is pointing towards the same functional surface of αC (Figs. 1 ▶ *a* and 4 ▶ *c*), mutations in which abolished the heterotypic interactions between IL-17RA and Act1 as well as IL-17-stimulated gene expression. Residue Tyr591 at the end of helix αG is only about a distance of 16 Å from Thr480 on helix αC. These observations imply that it is likely that the very C-terminal end of the SEFEX module could wind back to approach the close vicinity of helix αC, together forming a composite ligand-binding surface that is necessary for recruiting the adaptor protein Act1. Further structural studies will be necessary to provide a more in-depth understanding.

Although it has previously been reported that IL-17RC also requires a 20-amino-acid C-terminal extension to its SEFIR domain for function (Ho *et al.*, 2010 ▶), we believe that the intracellular signaling module of IL-17RC does not contain any extra functional motifs beyond its SEFIR domain. Ho and coworkers showed that truncation after residue 658 abolished IL-17 signaling, while constructs extending to at least residue 668 retained full function (Ho *et al.*, 2010 ▶). However, our structure-based sequence alignment indicates that the true C-terminal boundary for the IL-17RC SEFIR domain lies at around residue 663 (Zhang *et al.*, 2013 ▶). Therefore, truncation at residue 658 would cause partial deletion of the helix αE from the IL-17RC SEFIR domain, perturbing its structural integrity. However, the construct extending to residue 668 would retain the complete SEFIR structural module that is necessary for signaling.

**Figure 5:**
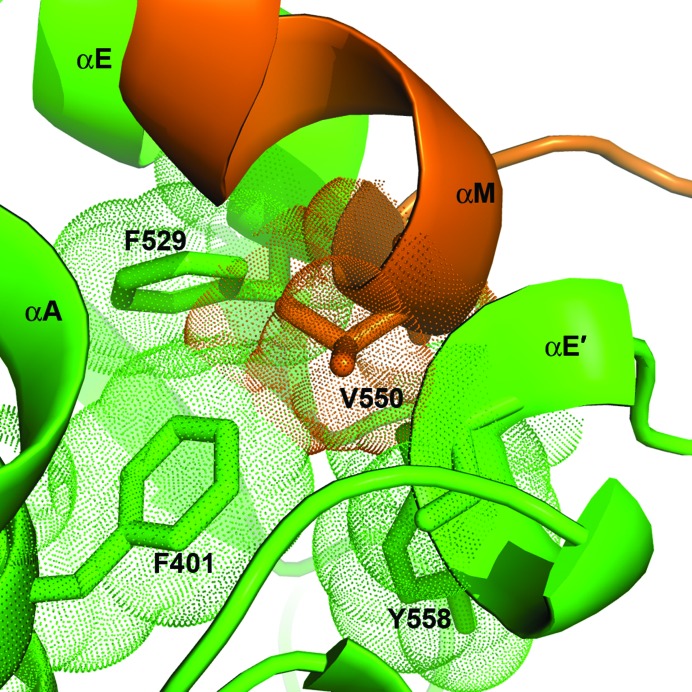
Val550 is a buried internal residue in IL-17RA SEFIR. The missing fragment (residues 541–555) connecting helices αE and αE′ of the crystal structure was modeled using *FALC-Loop* (http://falc-loop.seoklab.org/). Residue Val550 is located on helix αM and is buried inside the protein, making extensive hydrophobic contacts with three aromatic residues from helices αA (Phe401), αE (Phe529) and αE′ (Tyr558). The V550H mutation will disrupt the structural integrity.

Presumably owing to limited proteolysis treatment (§[Sec sec2]2), the fragment (residues 541–555 between helices αE and αE′) connecting the SEFIR domain and its C-extension is not visible in the structure of IL-17RA SEFIR. Our modeling showed that this missing region contains a short helix (αM) that is tightly packed against helices αA and αE (Fig. 5 ▶). It has previously been reported that a valine-to-histidine mutation in this helix (V550H) disrupted IL-17RA function (Maitra *et al.*, 2007 ▶), and this residue was therefore inferred to be a key functional residue. The current structural data suggest that this defective mutant is presumably owing to perturbation of the stability and structural integrity of IL-17RA. Specifically, residue Val550 is completely buried inside the protein and makes extensive hydrophobic contacts with three aromatic residues, Phe401 (αA), Phe529 (αE) and Tyr558 (αE′), playing a structural role rather than being involved in protein–protein interactions (Fig. 5 ▶).

## Supplementary Material

PDB reference: IL-17 receptor A SEFIR domain, 4nux

## Figures and Tables

**Table 1:** Statistics of data collection and refinement Values in parentheses are for the highest resolution shell.

	Se-SAD	Native
Data collection		
Beamline	19-ID, APS	19-ID, APS
Wavelength (Å)	0.97935	0.97935
Space group	*C*222_1_	*C*222_1_
Unit-cell parameters (Å)	*a* = 75.9, *b* = 133.8, *c* = 56.2	*a* = 76.2, *b* = 136.1, *c* = 55.4
Resolution (Å)	50–3.0	50–2.3
Total reflections	42331	87400
Unique reflections	6057	13104
*R*_merge_ † (%)	15.0 (78.3)	12.4 (65.8)
Multiplicity	7.0	6.7
Completeness (%)	99.9 (100)	98.0 (87.3)
〈*I*/σ(*I*)〉	12.8 (2.0)	15.3 (1.6)
Refinement		
Resolution range used (Å)		33.2–2.3
No. of reflections used		12124
*R*_work_/*R*_free_ ‡ (%)		18.9/24.1
R.m.s. deviations		
Bond lengths (Å)		0.008
Bond angles (°)		1.066
Ramachandran values (%)		
Preferred regions		96.3
Allowed regions		3.7

†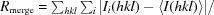 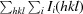.

‡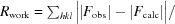 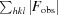; *R*_free_ was calculated using 5% of the data.
